# The Impact of Glycolysis and Its Inhibitors on the Immune Response to Inflammation and Autoimmunity

**DOI:** 10.3390/molecules29061298

**Published:** 2024-03-14

**Authors:** Beata Pająk, Rafał Zieliński, Waldemar Priebe

**Affiliations:** 1 Department of Medical Biology, Kaczkowski Military Institute of Hygiene and Epidemiology, Kozielska 4, 01-163 Warsaw, Poland; 2 WPD Pharmaceuticals, Żwirki i Wigury 101, 02-089 Warsaw, Poland; 3 Department of Experimental Therapeutics, The University of Texas MD Anderson Cancer Center, 1901 East Rd., Houston, TX 77054, USA; rjzielinski@mdanderson.org

**Keywords:** immune cells, inflammation, autoimmunity, glycolysis, glycolysis inhibitors, D-glucose, D-mannose, 2-deoxy-D-glucose

## Abstract

Glucose metabolism is a crucial biological pathway maintaining the activation of extra- and intracellular signaling pathways involved in the immune response. Immune cell stimulation via various environmental factors results in their activation and metabolic reprogramming to aerobic glycolysis. Different immune cells exhibit cell-type-specific metabolic patterns when performing their biological functions. Numerous published studies have shed more light on the importance of metabolic reprogramming in the immune system. Moreover, this knowledge is crucial for revealing new ways to target inflammatory pathologic states, such as autoimmunity and hyperinflammation. Here, we discuss the role of glycolysis in immune cell activity in physiological and pathological conditions, and the potential use of inhibitors of glycolysis for disease treatment.

## 1. Introduction

After glucose uptake, cytoplasmic glycolysis is the first step of glucose metabolism in eucaryotic cells, followed by oxidative phosphorylation (OXPHOS) within mitochondria. In physiological states, glycolysis generates 2 net adenosine triphosphate (ATP) molecules, whereas the OXPHOS pathway generates ~36 ATP molecules from 1 molecule of glucose [1].

Glycolysis is initiated by glucose phosphorylation via hexokinase (HK) to glucose-6-phosphate (G-6-P), which is further converted via phosphoglucose isomerase (PGI) into fructose 6-phosphate (F-6-P). Next, phosphofructokinase 1 (PFK1) phosphorylates F-6-P to fructose 1,6-diphosphate, which is further converted via aldolase into glyceraldehyde 3-phosphate. Subsequently, dehydrogenase converts glyceraldehyde 3-phosphate into 1,3-biphosphoglycerate, which is further converted into 3-phosphoglycerate, followed by 2-phosphoglycerate and phosphoenolpyruvate. Finally, pyruvate is synthesized. Pyruvate is a key molecule transported to mitochondria and converted into acetyl-CoA, which is then able to enter the Krebs cycle [2].

**Figure 1:**
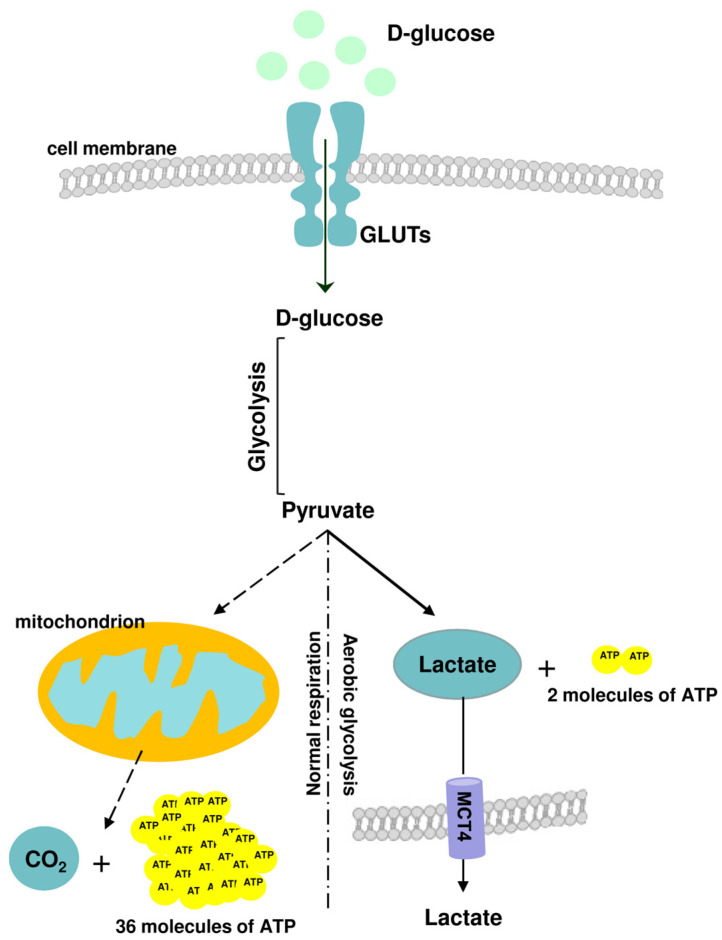
A brief outline of glucose metabolic pathways: normal respiration (oxidative phosphorylation, OXPHOS) and aerobic glycolysis. In physiological states, glycolysis generates 2 net adenosine triphosphate (ATP) molecules, whereas the OXPHOS pathway generates ~36 ATP molecules from 1 molecule of glucose.

In pathological states, such as oncogenesis or viral infection, cellular metabolism is altered and glycolysis, instead of OXPHOS, becomes the main glucose utilization pathway, which has been defined as a Warburg effect [3,4]. Importantly, the metabolism of immune cells has been discovered to be essential in controlling pro-inflammatory and anti-inflammatory profiles. Immune cells undergo metabolic reprogramming when they become activated [5]. During the peak of the inflammatory response, immune cells rely on glycolysis as a leading source of energy production, whereas during the resolution state, they prefer the OXPHOS pathway [5]. A brief outline of this metabolic shift is presented in Figure 1.

However, various immune cells and their subpopulations have distinct metabolism patterns with regard to glycolysis/OXPHOS ratio and/or metabolic substrate demands. On the other hand, the metabolic shift is also the main driver in unspecific immune cell activation leading to hyperinflammation and autoimmunity. Glycolysis modulation seems to be an attractive target for therapeutic intervention in such pathological states.

Herein, we summarize the role of glycolysis and metabolic reprogramming in immune cells in their physiological action, as well as the pathological states linked to hyperinflammation and autoimmunity. Further, the biological effects of glycolysis inhibitors and their potential role in hyperinflammatory response sequestration are discussed.

## 2. Metabolic Reprogramming of T Cells during Immune Responses

Naive T cells maintain low rates of glycolysis and predominantly oxidize pyruvate via OXPHOS or engage fatty acid oxidation (FAO) for ATP synthesis [6]. T cell activation via various antigens initiates their proliferation and differentiation into effector T cells (TEFF). The anabolic growth of activated T cells and daughter cell generation are linked to metabolism reprogramming, which is initiated via T cell receptor (TCR) binding. Activated cells upregulate glucose and amino-acid transporters, facilitating substrate uptake. Further, TCR mediates the upregulation of various transcription factors, such as c-Myc or estrogen-related receptor α (EERα), to enhance the intermediary metabolism. Growth factors and cytokines (including interleukin 2 (IL-2), IL-7, IL-15, and IL-21) released in response to antigen detection promote a metabolic switch via three main signaling pathways, phosphoinositol 3-kinase (PI-3K)/Akt, Janus kinase (JAK)/Signal Transducer and Activator of Transcription (STAT), or Mitogen-activated protein kinase (MEK)/Extracellular signal-regulated kinases (ERK) [7]. PI-3K/Akt further activates the mammalian target of rapamycin (mTOR), which then upregulates glycolytic enzyme activity and transmembrane glucose transporter expression [GLUTs] [8]. Moreover, T cell activation also triggers hypoxia-inducible factor 1α (HIF-1α) expression, even in the presence of oxygen [9]. As a result of the above-mentioned changes, activated CD4+ and CD8+ TEFF convert pyruvate into lactate, with or without oxygen. In addition to responding to extracellular signals, T cell metabolism is further modulated via intracellular metabolic changes. One example is the activation of mTOR kinase via increased levels of leucine and arginine because of the upregulated expression of the solute carrier (SLC) 7A5 transporter in activated T cells [10].

Another important example of how the metabolic status of T cells influences the signaling pathways that drive their differentiation is the regulation of adenosine monophosphate-activated protein kinase (AMPK) activity via increasing adenosine monophosphate (AMP) levels. AMPK promotes ATP synthesis and, at the same time, limits its consumption by inhibiting catabolic processes and mTOR action [11]. Metabolic changes have been described in all activated T cells, but it should be underlined that functionally distinct T cell subsets demonstrate different metabolic phenotypes and rely on distinct metabolic pathways to exert their specialist immune functions. For example, proinflammatory Th1, Th2, and Th17 subsets of helper CD4+ T cells are metabolically distinct from regulatory CD4+ T cells [12]. As reported by Shi et al. [12], Th1 and Th17 cells are characterized by a highly glycolytic metabolism; additionally, Th17 cells are glutamine-dependent. On the other hand, regulatory T cells tolerate low glucose concentrations and utilize fatty oxidation processes to maintain their biological functions [13].

When the antigen is removed, the majority of TEFFs are eliminated. Only a small population of long-lived antigen-specific memory T cells (TM) remain in the body. TM metabolism is similar to that of native T cells, with dominant OXPHOS and a low nutrient uptake. Although higher reliance on OXPHOS is a common metabolic trait of TM cells, various TM cell subpopulations (central memory T (TCM) cells, tissue-resident memory T (TRM) cells, and effector memory T (TEM) cells) differ in terms of OXPHOS/glycolysis ratios, as well as substrate utilization. TEM cells rely less on OXPHOS than TCM or TRM cells. Moreover, TCM cells preferentially use exogenous fatty acids for mitochondrial respiration, whereas TM cells utilize glucose, glutamine, and fatty acids [14]. Importantly, TM cells maintain an increased mitochondria mass, which serves as an additional mitochondrial spare respiratory capacity (SRC) in the case of their engagement in an immune response to secondary TCR activation [15].

## 3. Metabolic Reprogramming of B Cells during Immune Responses

Naive quiescence B cells exhibit a low basal metabolism that is significantly upregulated in response to antigen presentation. Activated B cells accumulate nutrients and increase their biomass to prepare for an increasing cell size and rapid proliferation. It has been demonstrated that distinct phases of the humoral immune response demand modifications of the B cell metabolic state to match their biological function. An interesting feature of B cell metabolism is that its reprogramming is not exclusively based on glucose consumption and glycolysis activation, unlike T cells. In the case of B cells, glycolysis is only one of multiple metabolic routes modulated during the immune response [16].

Upon activation and clonal expansion, B cells increase their glucose uptake and its breakdown through glycolysis. It has been shown that antigen stimulation induces the expression of glucose transporters (GLUTs 1, 4, 8, and 11) [17]. Glycolytic metabolism is further supported by the activation of the HIF-1α, PI-3K/Akt, and IL-4/STAT6 signaling pathways. On the other hand, glycolysis activation in B cells is not followed by OXPHOS shutting down. The upregulated glycolysis delivers a higher amount of pyruvate for oxidative phosphorylation. Further, other substrates, such as glutamine and fatty acids, are incorporated into OXPHOS [18].

## 4. Metabolic Reprogramming of Macrophages during Immune Responses

Macrophages are another highly heterogeneous immune cell population with various important functions in the innate immune response. The broad spectrum of macrophage actions results from their plasticity in modifying and specializing their properties in response to environmental stimuli. This process is defined as macrophage polarization [19]. In general, macrophages can play a pro-inflammatory (M1) or anti-inflammatory (M2) role, depending on the cell surface markers they encounter and their secreted cytokines profile. M1 pro-inflammatory macrophages mainly use glycolysis and the pentose phosphate pathways (PPP) to synthesize ATP, whereas the Krebs cycle is disrupted and OXPHOS and FAO processes are downregulated [20]. Similarly to T cells, metabolic reprogramming in M1 macrophages is initiated by activating the HIF-1α transcription factor. As expected, HIF-1α upregulates the expression of GLUT1 glucose transporters and glycolytic enzymes, facilitating rapid glucose uptake and glycolysis [19]. Moreover, the glycolytic phenotype of M1 macrophages is supported via the 6-phosphofructo-2-kinase B (PFKFB) and the pyruvate kinase M2 (PKM2) enzymes’ isoforms. The PFKFB3 isoform is predominantly expressed in M1 macrophages, and when compared to other PFKFB isoforms, thereby less efficiently catalyzes the conversion of fructose-2,6-bisphosphate into fructose 6-phosphate, enhancing the glycolytic flux [19]. The PKM2 isoform additionally stimulates HIF-1α-dependent gene transcription [21].

The role of glycolysis in M2 macrophage function is still unclear. Reports have shown that the activation of glycolysis occurs in M2 macrophages, and its inhibition affects M2 polarization and biological activity. On the other hand, several reports have suggested that glycolysis is not crucial for M2 action, as long as OXPHOS and FAO remain intact [22]. Contradictory to M1, in M2 macrophages, the PFKFB1, not B3, isoform effectively converts fructose-2,6-bisphosphate into fructose-6-phosphate, lowering the glycolytic rate [23]. Altogether, these data demonstrate that M2 macrophages are more flexible in their metabolism.

## 5. Metabolic Reprogramming of Dendritic Cells during Immune Responses

Dendritic cells (DCs) are antigen-presenting cells that bridge innate and adaptive immune responses. In the presence of a pathogen, DCs activate several innate immune response components, such as pattern recognition receptors (PRRs), intracellular signaling pathways, the synthesis of antimicrobial mediators, and inflammatory cytokine secretion. On the other hand, DCs process and present antigens to T cells, which activates the adaptive immune response [24]. It is known that DCs rely on a tightly regulated metabolism to activate an immune response or promote tolerance. Unfortunately, due to the limitations of in vitro and in vivo models, there are still gaps in our understanding of DC metabolism that remain to be explained.

While the glycolytic shift is a hallmark of murine bone-marrow-derived DC (BMDC) activation, this phenomenon is not obvious in human DCs. Available reports indicate that context-specific metabolic reprogramming determines changes in immature, steady-state, inflammatory activation and the initiation of immune tolerance in different microenvironmental and physiological states [25]. Quiescent DCs reside mostly in peripheral tissues and utilize glucose to create pyruvate, which is metabolized in the OXPHOS process. ATP synthesis also occurs through fatty acid conversion. The key regulator of quiescent DC metabolism is the AMPK/mTOR pathway [26]. On the other hand, as reported recently by Moller et al. [27], an immediate shift to glycolytic metabolism is a conserved response to PRR signaling, such as toll-like receptors’ (TLR) or C-type lectin receptors’ (CLRs) activation. Acute glycolytic activation influences several intracellular processes, such as fatty acid synthesis and mitochondrial trafficking and activity, that are vital for proper DC function [28].

Future studies on how various environmental factors influence DC metabolism and epigenetics may lead to an advanced understanding of DCs’ biology and the role of their metabolic adaptation in immunity and inflammation.

**Figure 2:**
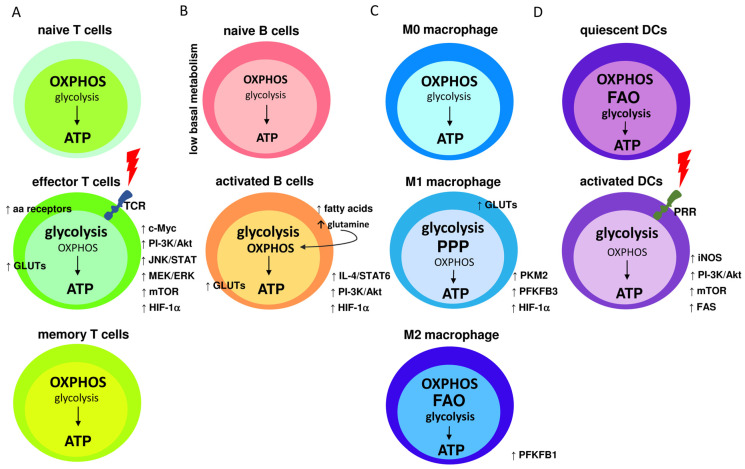
Metabolic characteristics of naive and activated immune cells; (**A**) naive T cells maintain low rates of glycolysis and predominantly oxidize pyruvate via OXPHOS or engage fatty acid oxidation (FAO) for ATP synthesis. TCR receptor binding triggers T cells’ activation, leading to elevated levels of transmembrane glucose (GLUTs) and amino acids (aa) receptors. TCR signaling also upregulates c-Myc, PI-3K/Akt, JNK/STAT, MEK/ERK, mTOR, and HIF-1α. Consequently, glycolysis becomes the dominant pathway for glucose utilization. In memory T cells, glycolysis rate is downregulated and OXPHOS is the main source of ATP synthesis; (**B**) naive quiescence B cells exhibit low basal metabolism, with the main participation of the OXPHOS cycle. Activated B cells increase glycolysis rate, which is followed by elevated levels of GLUTs. Glycolytic metabolism is further supported by the activation of HIF-1α, PI-3K/Akt, and IL-4/STAT6 signaling pathways. Regardless of the high glycolysis rate, OHPHOS is still active and incorporates glutamine and fatty acids for more robust ATP synthesis; (**C**) under normal conditions, naive M0 macrophages obtain energy by efficiently employing OXPHOS. M1 pro-inflammatory macrophages mainly use glycolysis and the pentose phosphate pathways (PPP) to synthesize ATP, whereas the Krebs cycle is disrupted and OXPHOS and FAO processes are downregulated. Metabolic reprogramming activates HIF-1α and GLUTs expression. Also, the levels of the glycolytic enzymes PFKFB3 and PKM2 are elevated. M2 activated macrophages use FAO and OXPHOS as the main metabolic pathways while increasing glucose utilization, however, glycolysis is also elevated in comparison to M0 macrophages; and (**D**) quiescent DC cells utilize glucose to create pyruvate, which is metabolized in the OXPHOS process. ATP synthesis also occurs through FAO. Upon PRR receptor activation, PI-3K/Akt pathway is activated, leading to increased fatty acids synthesis (FAS) and mTOR activity. PRR activation also induces nitric oxide (NO) production by inducible NO synthase, which stimulates the glycolysis pathway under decreased OXPHOS.

A summary of the metabolic characteristics of the naive and activated immune cells described above is illustrated in Figure 2.

## 6. Hyper-Inflammatory Response—The Potential of Glycolysis Inhibition

As described above, immune system activation requires a metabolic shift of immune cells to execute their activity quickly and effectively. However, this is also true in the case of unspecific immune activation, such as during the course of autoimmune diseases. Metabolic reprogramming is a crucial process that fuels overactive immune cells [29]. In the case of severe viral infection and the so-called “cytokine storm”, a potentially life-threatening process, glycolysis plays a crucial role in the hyper-inflammatory response [30]. The ability to modulate the glycolysis process (to inhibit or stimulate its processing) could be useful for immune response control in various physiological states. Thus, several reports have examined the efficacy of several glycolysis inhibitors as anti-inflammatory agents across multiple disease models.

## 7. 2-Deoxy-D-glucose as an Anti-Inflammatory Agent

One of the most broadly tested glycolysis inhibitors is D-glucose analog 2-deoxy-D-glucose (2-DG), lacking the -OH group in the C-2 position in the pyranose ring [30]. 2-DG competes with glucose to bind to transmembrane GLUTs and enter cells. Next, 2-DG undergoes phosphorylation via hexokinase to 2-deoxy-D-glucose-6-phosphate (2-DG-6P) and is trapped inside the cells. 2-DG-6P cannot be further metabolized via phosphofructoisomerase, thus, the glycolysis pathway is inhibited [31]. Numerous anticancer and antiviral studies have described the beneficial effects of glycolysis inhibition with 2-DG [31,32]. However, its anti-inflammatory properties are less well known.

Zhao et al. [33] showed that 2-DG can inhibit M2 macrophage polarization in a dose-dependent manner, thus preventing immune response activation in response to chitin treatment in an in vivo model. Moreover, in an ovalbumin (OVA)-induced allergic airway inflammatory mouse model, two-day 2-DG pretreatment significantly decreased M2 macrophage activation and prevented the pathogenesis of asthma. Similarly, Cai et al. [34] found that 2-DG treatment limits adjuvant arthritis (AA) development. The authors demonstrated that 2-DG downregulated the arthritis index and alleviated cellular infiltration, synovial hyperplasia, and bone erosion in AA rats. With regard to immune cells, it appeared that 2-DG affected peritoneal macrophage polarization via increasing the arginase1 (Arg1) level and a concomitant reduction in inducible nitric oxide synthase (iNOS). 2-DG also activated the AMPK via phosphorylation and reduced the activation of nuclear factor κB (NF-κB) in the AA rats’ peritoneal macrophages, which was crucial for macrophage activation [34]. 

The influence of 2-DG on macrophage polarization was also examined by Wang et al. [35]. The authors found that 2-DG-mediated glycolysis inhibition and also impaired the oxidative phosphorylation process, manifested by a significant reduction in ^13^C-labeled Krebs cycle metabolites and intracellular ATP levels. Further, 2-DG treatment inhibited JAK/STAT6 signaling pathway activity. Interestingly, suppressed glycolysis and reduced pyruvate production did not affect OXPHOS or alternative M2 macrophage activation. According to a more detailed analysis, the authors concluded that the observed 2-DG effects on M2 macrophages may not be attributed solely to its effects on glycolysis, because glucose depletion did not exert a similar response and had no effect on M2 macrophage differentiation. Wang et al. [35] postulated that the main driver of impaired JAK/STAT6 activity is decreased ATP levels and ATP/ADP ratio but increased AMP/ATP ratio, whereas glucose depletion does not influence ATP production. In summary, 2-DG at commonly used doses [10 mM] inhibits glycolysis and OXPHOS, thereby suppressing ATP production, JAK-STAT6 pathway activation, and the M2 phenotype. The authors concluded that, in macrophages, 2-DG cannot be confirmed as a specific glycolysis inhibitor. The authors did not hypothesize about other possible intracellular 2-DG targets, which could potentially explain its biological action. Based on our knowledge about 2-DG action, the important intracellular process that could be affected by 2-DG presence is protein glycosylation, crucial for the proper biological function of intracellular proteins.

### 7.1. The Importance of 2-Deoxy-D-glucose Interreference with Protein Glycosylation

The anti-inflammatory 2-DG properties are not only based on glycolysis inhibition, but also on its ability to interfere with the D-mannose metabolic pathway. Historically, 2-DG was synthesized from D-glucose by eliminating the hydroxyl group at C-2. However, removing the hydroxyl group at C-2 in the D-mannose molecule leads to the same 2-DG compound. Thus, 2-DG can interfere with both the D-glucose and D-mannose pathways. Altering mannose-related intracellular conversion leads to dysregulation of the *N*-glycosylation process, a crucial cellular process for protein maturation [32]. 

It should be noted that most cell surface and secreted proteins involved in the immune response are glycosylated. Numerous studies have reported that glycosylation influences the function of all immune cells [36]. Glycans play a crucial role in intercellular contacts and leukocyte migration. These interactions are essential in the activation and proliferation of leukocytes and during the immune response [37]. Key immune proteins, such as T-cell receptors (TCRs), major histocompatibility complex (MHC), toll-like receptors (TLR), and antibodies, are glycosylated. Furthermore, oligomerization and glycosylation processes can alter cytokine functions that orchestrate immune functions. It is theorized that the cell uses the glycosylation of cytokines to alter cytokines’ functions. One postulated possibility is that multiple glycosylation forms compete for receptor binding, resulting in a natural selection process or possibly antagonistic effects [38].

Inflammation is known to modify the glycosylation pattern of glycolipids and glycoproteins. It is well known that the glycosylation of acute-phase proteins changes during acute and chronic inflammation [39]. Moreover, chronic inflammation and prolonged exposure to proinflammatory cytokines have been shown to modulate the substrate synthesis pathways, as well as the expression of glycosyltransferases required for the biosynthesis of N-glycans. The resulting N-glycosylation changes can further contribute to disease pathogenesis by modulating various aspects of immune cell processes [40].

Current anti-inflammatory therapies are based on non-steroidal anti-inflammatory drugs (NSAIDs) and corticosteroids, which are still the most effective agents for controlling the clinical manifestations of inflammation. However, they usually need to be used with other “disease-modifying” strategies to control chronic inflammatory disorders effectively [41]. Recently, anti-proinflammatory cytokine therapies against interleukin (IL)-6, tumor necrosis factor (TNF)-α, and IL-1 were introduced in inflammatory disease therapy, especially for rheumatoid arthritis [42]. Such therapies are mainly performed using monoclonal antibodies against cytokines or cytokine receptors. Anti-IL-6 drugs (tocilizumab and sarilumab) were also highly effective in the treatment of medium to severe forms of COVID-19 pneumonia, reducing the risk of mortality due to multi-organ failure [43]. While anti-proinflammatory cytokine therapies such as mAb and soluble cytokine receptors are clinically effective, these protein therapies require multiple injections. They are also expensive, hindering their use as the primary choice to treat various inflammatory diseases. In practical terms, orally bioavailable and easy-to-synthesize drugs directed against diverse proinflammatory cytokine systems would be preferable. In this regard, 2-DG is a promising anti-inflammatory compound.

Recent studies published by Uehara et al. [42] showed that 2-DG significantly attenuated the proinflammatory response to IL-6 by inhibiting IL-6R gp130 glycosylation. 2-DG also blocked signals induced by TNF-α, IL-1β, and interferon (IFN)-γ, efficiently alleviating a mouse model of inflammatory bowel disease and human rheumatoid arthritis. Further, 2-DG also attenuated lipopolysaccharide (LPS)-induced pulmonary inflammatory responses in a mouse model of a cytokine storm and Acute Respiratory Distress Syndrome (ARDS) [42]. The authors concluded that the N-glycosylation of proinflammatory cytokine receptors is an unrecognized potential target for anti-inflammatory therapies. However, they also pointed out several disadvantages of using 2-DG, such as toxicity and low pharmacological specificity, which may limit its use in treating inflammatory diseases. The authors claimed they could not pinpoint the possible mechanism of N-glycosylation disturbances in response to 2-DG. However, bearing in mind that 2-DG is able to affect the mannose metabolic pathway, its influence on the glycosylation pattern is not surprising to us. Based on the available data, the authors did not identify, other than cytokine receptors, the types of proteins affected by 2-DG. They concluded that, due to the rapid 2-DG metabolism, only a limited number of proteins may be affected by once-daily 2-DG administration [42]. As far as we know, the above paper has the only available data documenting 2-DG-induced glycosylation modulation as an anti-inflammatory strategy. Overall, these promising data should lead to further exploration of 2-DG’s impact on inflammation and glycosylation processes.

### 7.2. 2-Deoxy-2-(^18^F)fluoro-D-glucose (^18^FDG) as a Tracker for Inflammation and Infection

Positron emission tomography (PET) is a non-invasive imaging technique widely used for the diagnosis, staging, and monitoring of responses to therapy in oncological patients. The standard PET radiotracker is the radiolabeled fluorine 2-DG analog FDG, introduced in 1976 [44]. Similar to 2-DG, FDG is transported via glucose transporters competing with glucose. The discovery in 1980 that FDG accumulates in glycolysis-elevated cancer cells first introduced FGD into clinics [45]. However, the discovery of the upregulated glycolysis process in activated immune cells opened up new avenues for PET as a tool for inflammation diagnosis. As a result, Guidelines for ^18^F-FDG Use in Inflammation and Infection were published for use in Europe and the United States [46]. It should be emphasized that more data than those currently available are needed in order to advance PET imaging as a first-line diagnostic tool. However, based on a cumulated reported accuracy analysis, ^18^F-FDG PET could be applied for sarcoidosis [47], peripheral bone osteomyelitis [48], suspected spinal infections, and fever of unknown origin (FUO) diagnoses [49]. Moreover, an evaluation of metastatic infection of high-risk patients with bacteremia [50] and primary evaluation of vasculitides [51] could be performed. Other well-described applications, but without sufficient evidence-based indications, include the following: evaluation of potentially infected liver and kidney cysts in polycystic disease [52], suspected infection of intravascular devices [53], AIDS-associated opportunistic infections and associated tumors [54], and assessment of metabolic activity in tuberculosis lesions [55]. Recent data also indicate other indications for FDG-PET imaging, like rheumatologic diseases (rheumatoid arthritis, rheumatic polymyalgia, and lupus erythematosus) [56], osteoporosis [57], spondylodiscitis [58], and idiopathic retroperitoneal fibrosis (RPF) [59].

We expect that the list of inflammatory diseases that can be diagnosed with FDG-PET will be extended in the future. Along with standard diagnostic techniques, identifying highly active immune cell lesions due to their high glycolysis rate significantly benefits proper patient management and their outcomes.

## 8. 2-DG as a Drug-Candidate

2-DG’s ability to affect glycolysis and protein glycosylation makes it an efficient cytotoxic agent against highly glycolytic cells. As mentioned, metabolic shift is characteristic of viral infection and cancer cells. Furthermore, the described data indicate that the glycolysis process is necessary for immune cell action in both physiological and pathological conditions. Importantly, 2-DG effects were primarily observed in glycolytic cells, without a notable influence on normal cells [60]. Thus, 2-DG has been explored as a cytotoxic compound and an adjuvant for various clinically used chemotherapeutic anticancer drugs [31]. To date, 2-DG has been tested in several oncological clinical trials. Further, the recent SARS-CoV-2 pandemic introduced 2-DG as a viral indication when COVID-19 patients were treated as part of a phase II study [61].

Despite the numerous preclinical and clinical studies, the use of 2-DG in clinics is still limited. The main disadvantages of 2-DG are its rapid metabolism and short half-life, which make 2-DG a poor drug candidate [62]. Moreover, 2-DG competes with blood glucose for cellular GLUTs, thus, it must be used at relatively high concentrations (≥5 mmol/L) [63]. However, 2-DG also has promising characteristics that were documented in clinical studies. As reported by Stein et al. [64], 14-day administration of 2-DG (45 mg/kg) was defined as safe, and no dose-limiting toxicities were observed. Further, an article describing the use of 2-DG in humans was published by Raez et al. [65]. In this study, based on the overall tolerability of the 2-DG treatment, a dosage of 63 mg/kg was categorized as safe. At a higher dose of 88 mg/kg, mild side effects were observed, such as elevated plasma glucose levels (>300 mg/dL) and glucopenia symptoms, including sweating, dizziness, and nausea [65]. Moreover, gastrointestinal bleeding and reversible grade 3 QTc prolongation were noted. In 2022, the results of the phase II study of 2-DG in COVID-19 patients were published. Patients were randomized to receive 63, 90, or 126 mg/kg/day 2-DG, in addition to standard-of-care (SOC) [61]. The results corresponded with the previous reports, as all three dose levels of 2-DG were well tolerated. Although adverse events were reported in 33 (30.3%) patients, most (86%) were classified as mild. The most common side effect was hyperglycemia. No clinically significant cardiac QT interval prolongations were reported in the 2-DG treatment arm [61]. Unfortunately, as of today, no clinical observations regarding the activity of immune cells in response to 2-DG have been monitored. Furthermore, studies verifying the anti-inflammatory potential of 2-DG are also limited.

Overall, 2-DG is a promising molecule with multitargeted potential and a good tolerability profile. Nevertheless, poor pharmacokinetic properties and possible side effects warrant a search for other molecules that target the same metabolic pathway, but can overcome the 2-DG limitations described above. One possible solution is the identification of novel 2-DG derivatives or prodrugs, which would have improved drug-like properties.

### Novel 2-DG Analogs and Their Potential for Immunomodulatory Applications

**Figure 3:**
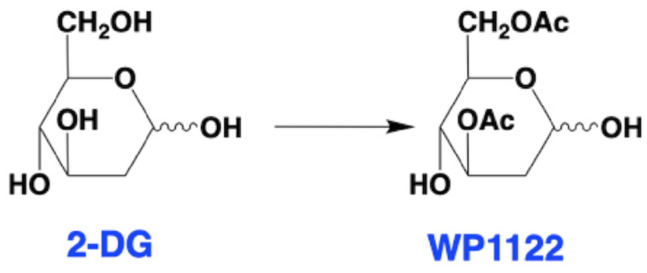
Structures of 2-DG and WP1122. Within the cell, WP1122 is deacetylated by cellular esterases and release active 2-DG.

It was previously shown that even small changes to a chemical structure can profoundly affect the pharmacokinetic properties of the molecule. A good example is acetylation, which modulates membrane permeability and allows acetylated molecules for intracellular uptake via passive diffusion. In contrast, charged compounds could cross the membrane only via a specific transporter [66]. Fokt et al. [67] hypothesized that the acetylation of 2-DG, leading to a prodrug, could improve 2-DG’s drug-like properties. The authors prepared stable mono- and di-acetates of 2-DG with significant water solubility. Among the tested acetyl 2-DG derivatives, 3,6-di-*O*-acetyl-2-deoxy-D-glucose (WP1122) has been identified as a potent glycolysis inhibitor (Figure 3).

Further studies have shown that, in contrast to 2-DG, WP1122 enters the cells via passive diffusion without GLUTs contribution [68]. Diacetylated WP1122 is metabolized via intracellular esterases to monoacetate-2-DG analogs, followed by active 2-DG molecule release. As demonstrated by in vivo studies, the inhibitory effect of WP1122 is 2–10 times stronger than 2-DG alone [69]. Thus, WP1122 has been verified as an efficient anticancer agent [70], however, its immunomodulatory activity has yet to be explored.

## 9. Other Glycolysis Inhibitors and Their Anti-Inflammatory Potential

Although 2-DG is the most well-known glycolysis inhibitor that has been examined in numerous studies, other compounds able to inhibit glycolysis should also be highlighted as potential anti-inflammatory drugs.

### 9.1. 4-Octyl Itaconate

Liao et al. [71] reported that 4-Octyl itaconate (4-OI) is able to inhibit glycolysis in activated macrophages, thus exerting anti-inflammatory effects. 4-OI is a cell-permeable itaconate derivative that exerts thiol reactivity. It was previously shown that 4-OI alkylated cysteine residues on kelch-like ECH-associated protein 1 (KEAP1) and then activated nuclear factor (erythroid-derived 2)-related factor 2 (Nrf2) to exert antioxidant and anti-inflammatory effects in an LPS-stimulated mouse model [72]. Liao et al. [71] also found that 4-OI induced a posttranslational modification of Cys 22 of glyceraldehyde 3-phosphate dehydrogenase (GAPDH), reducing its enzymatic activity and, consequently, aerobic glycolysis rate. In response to 4-OI treatment, macrophage activation induced by LPS was significantly diminished, as confirmed via an ELISA measurement of IL-1β released by macrophages.

Other studies published by Deng et al. [73] confirmed that 4-OI blocks glycolysis via GAPDH inhibition and, thus, it is able to diminish the proinflammatory activity of CD4+ and CD8+ T cells. The authors also revealed that 4-OI did not affect the viability of resting T cells, but preferentially modulated activated T cell-derived cytokine levels. The authors concluded that 4-OI could be a potential therapeutic agent in autoimmune diseases and Graft-Versus-Host Disease (GVHD) [73].

### 9.2. (E)-1-(Pyridin-4-yl)-3-(quinolin-2-yl)prop-2-en-1-one (PFK15)

6-phosphofructo-2-kinase/fructose-2,6-biphosphatase (PFKFB3) is the key regulator of glycolytic flux, which catalyzes the synthesis of fructose-2,6-bisphosphate (6P2). This activates phosphofructokinase 1 (PFK1), the rate-limiting key enzyme of glycolysis [74]. PFK15 is a small molecule inhibitor of PFKFB3 activity, decreasing glucose uptake and lactate production due to glycolysis inhibition [75]. Several reports support the potent anticancer activity of PFK15 [76], but its biological effects are also related to limiting the activation of immune cells in chronic infections and autoimmune diseases. Martins et al. [77] found that PFK15 treatment reduced metabolic reprogramming in T cells, delaying the onset of type I diabetes. On the other hand, Mangal et al. [78] demonstrated that PFK15 is a potent inhibitor of LPS-stimulated bone-marrow-derived DCs (BMDCs). Furthermore, in vivo experiments with a collagen-induced arthritis (CIA) mouse model showed that PFK15 delivery using microparticles with alpha-ketoglutarate (aKG) in the polymer backbone (paKG MPs) generated immunosuppressive antigen-specific T cell responses. Based on these results, the authors hypothesized that sustained glycolytic inhibition of DCs in the presence of an antigen can induce antigen-specific immunosuppressive responses, providing a strategy potentially applicable to treating autoimmune diseases [77].

Another example of the anti-inflammatory effects of glycolysis inhibition via PFK15 was described recently by Zhou et al. [79]. A microarray analysis of inflammatory bowel disease (IBD) patient-derived fibroblasts revealed the elevated expression of *PFKFB3* in inflamed versus non-inflamed tissues from IBD patients. Next, a Seahorse real-time cell metabolic analysis demonstrated a significantly higher extracellular acidification rate and lower oxygen consumption in inflamed fibroblasts, indicating an elevated glycolysis pathway [79]. To evaluate the effects of PFKFB3 inhibition and glycolysis downregulation for IBD-related inflammation, IBD mice were treated with PFK15 inhibitor (25 mg/kg every three days). The endoscopic colitis score was assessed after 8 days of the experiment, and colon tissue was histologically examined. The authors found that the presence of PFK15 reduced the histology score in colitis mice. Additionally, an immunohistochemical analysis showed a decreased infiltration of CD45+ cells and lower expression of chemokines like TNF-α, IL-1β, and CXCL5, 9, and 12.

Finally, the inhibition of PFKFB3 and glycolysis by PFK15 has been reported to alleviate inflammation and suppress the growth of various tumors in mice, thus, its clinical introduction is highly desired. The PFK15-based synthetic molecule, PFK158, is currently in a phase I clinical trial (NCT02044861) [80] for patients with advanced solid tumors.

### 9.3. 3-(3-Pyridinyl)-1-(4-pyridinyl)-2-propen-1-one (3PO)

3-(3-Pyridinyl)-1-(4-pyridinyl)-2-propen-1-one (3PO) is another PFKFB3 kinase inhibitor that has been shown to effectively decrease the enzymatic reactions of the glycolytic pathway [81]. 3PO has been tested in several cancer models, including melanoma [82], lung cancer, glioblastoma, colon adenocarcinoma, pancreatic cancer [83], and others, as a glycolysis inhibitor and potent cytotoxic compound. 3PO’s effects also verified it as an anti-inflammatory agent in, for example, a sepsis-related acute lung injury (ALI) model [84]. Gong et al. [84] examined 3PO’s effects in a cecal ligation and puncture (CLP)-induced ALI mouse model. 3PO treatment after the CLP procedure significantly improved the animal survival rate and decreased lung inflammation, histopathological changes, and lung apoptosis [84]. Further, in an in vitro model of LPS-stimulated human alveolar epithelial A549 cells, 3PO’s presence attenuated LPS-induced cell apoptosis, inflammatory cytokine production, and ROS generation [84].

Positive 3PO effects have also been reported in rheumatoid arthritis (RA) research [85]. The authors found that, in the RA model, TLR2 receptor activation induced metabolic changes in RA synovial fibroblasts (RASFC), leading to proinflammatory cytokine production, cell migration/invasion, and the transcriptional upregulation of nuclear factor kappa B (NF-κB) and STAT3 phosphorylation [85]. 3PO treatment [20 μM] significantly inhibited the above-mentioned changes, exerting a protective effect.

Anti-inflammatory effects of 3PO were also reported in atherogenic models induced by an oxidized low-density lipoprotein (oxLDL)-induced process called trained immunity [86], atherosclerotic macrophages [87], synovial inflammation [88], and others.

An interesting study concerning 3PO action was reported recently by Wik et al. [89]. The authors examined the effects of 3PO in the chronic inflammation of endothelial cells using an in vitro HUVEC cell line model. They found that 3PO pretreatment [20 μM] caused a rapid reduction in IL-1β- and TNF-mediated NF-κB activation, as well as c-Jun N-terminal kinase (JNK). However, when 3PO was replaced with an alternative PFKFB3 inhibitor, either 7,8-dihydroxy-3-(4-hydroxy-phenyl)-chromen-4-one (YN1) or shRNA-mediated silencing of PFKFB3, the prevention of cytokine-induced NF-κB signaling and upregulation of the adhesion molecules VCAM-1 and E-selectin was observed [89]. These results indicate that the anti-inflammatory action of 3PO in human endothelial cells is not limited to the inhibition of PFKFB3.

On the other hand, isothermal titration calorimetry results published by Veseli et al. [90] revealed that 3PO did not bind directly to PFKFB3, even up to a 750 µm concentration, in contrast to 3 µm of AZ67, another potent and specific PFKFB3 inhibitor. The authors confirmed that 3PO can accumulate inside the cells, leading to a pH decrease and the subsequent inhibition of glycolytic enzymes, but these cellular effects are not directly connected to PFKFB3 activity [90]. The authors suggested that the cellular targets of 3PO are rather monocarboxylate transporters 1 and 4 (MCT1 and MCT4) [90], but this hypothesis needs to be further explored.

### 9.4. Dichloroacetate (DCA)

Dichloroacetate (DCA) inhibits pyruvate dehydrogenase kinase (PDK), leading to pyruvate dehydrogenase (PDH) activation and promoting oxidative phosphorylation [91]. DCA has been used in humans for decades to treat acquired and congenital forms of lactic acidosis by shifting pyruvate metabolism from cytoplasmic lactate production to the oxidative production of acetyl-CoA in the mitochondria [92]. As an efficient aerobic glycolysis inhibitor, DCA has also been tested in several cancer models as a cytotoxic agent, however, its ability to inhibit glycolysis could also be beneficial in inflammatory states.

As reported by Bian et al. [93], DCA significantly improved the outcome of animals with collagen type II (CII)-induced arthritis (CIA), representing human rheumatoid arthritis. According to the presented data, mice treated with DCA displayed a delayed onset of CIA with a lower severity and frequency of arthritis. Further, DCA administration decreased cartilage and joint destruction and arthritis-induced cortical bone mineral loss. Interestingly, only female mice benefited from DCA treatment due to the presence of estrogen. However, the detailed mechanisms linking the cell metabolism to estrogen activity in the CIA model have not been explained [93]. The positive effects of DCA treatment were also reported in the course of sepsis [94], atherosclerosis [95], and colitis [96].

Due to its promising cytotoxic action in cancer cells, DCA received orphan drug status. According to the available data, DCA treatment does not have severe side effects and there is no development of a drug resistance phenotype. Moreover, DCA is active preferentially on glycolysis-elevated malignant cells and does not affect the proliferation and apoptosis of normal cells [97]. However, comparing DCA to the other commercially available drugs, its therapeutic dosage is very high, thus, novel DCA analogs, such as N-phenyl dichloroacetamide [98] or *N*-aryl-2,2-dichloroacetamide [99], have been synthesized to improve DCA bioavailability. Their anti-inflammatory potential has not been tested in clinical trials yet.

### 9.5. 3-Bromopyruvate (BrPA)

3-Bromopyruvate (BrPA) is a pyruvate and lactate analog able to inhibit key glycolysis enzymes, such as hexokinase II (HKII), glyceraldehyde-3-phosphate dehydrogenase, and lactate dehydrogenase (LDH) [100]. BrPA effectively inhibits the glycolysis process, so was considered to be an efficient anticancer compound. It was tested in clinical trials to treat patients with fibrolamellar hepatocellular carcinoma (HCC) [101].

The anti-inflammatory action of BrPa has also been recognized. Okano et al. [102] demonstrated that BrPA treatment significantly decreased the arthritis and histological scores in SKG mice. Moreover, changes in immune cells were observed, such as an increase in T_reg_ cells, a decrease in Th_17_ cells, and a decrease in activated DCs in the spleen. The BrPA effect was also confirmed with in vitro studies, where BrPA facilitated the differentiation of T_reg_ cells, suppressed Th17 cells, and inhibited the activation of DCs [93]. Only a limited number of available publications describe the anti-inflammatory use of BrPA, however, the data from Okano et al. [102] represent another strong argument for considering glycolysis inhibition as a potent anti-inflammatory strategy.

### 9.6. Small Molecules Inhibitors of GLUTs

As elevated glycolysis makes activated cells highly dependent on efficient glucose delivery, the inhibition of GLUTs activity is a rational strategy for glycolysis downregulation. As reported recently by Chen et al. [103], the GLUT1 inhibitor BAY-876 effectively downregulated activated CD4+ T cells and macrophages by 41% and 15%, respectively. GLUT1 inhibition also reduced CD4+ T cell proliferation and IFN-γ secretion by 20%. Moreover, TNF-α secretion from macrophages was reduced by 27% [103].

Further, Li et al. [104] demonstrated that GLUTs’ inhibition could be a positive therapeutic strategy in lupus treatment. Using a CD-5 inhibitor, the authors downregulated glycolysis in activated CD4+ T cells while promoting fatty acid oxidation and the pentose phosphate pathway. Moreover, CG-5 diminished Th1 and Th17 polarization and enhanced T_reg_ differentiation. Importantly, in vivo data demonstrated that CG-5 administration ameliorated lupus phenotypes in both spontaneous and induced models of lupus. Finally, CG-5 was also effective in human CD4+ T cells [104]. According to the rationale described by Di Dedda et al. [105], GLUTs activity limitation, especially GLUT1, could also be an avenue in type 1 diabetes therapy.

It should be noted that numerous studies with GLUTs inhibitors have been performed for oncological indications, where glycolysis inhibition was a powerful cytotoxic strategy. Taking into consideration the mechanisms described above and available results in autoimmunity models, their application in autoimmune disease treatment requires further investigation.

### 9.7. Koningic Acid (Heptelidic Acid)

Koningic acid is a potent inhibitor of GAPDH, which binds to the cysteine residue at the active site [106]. GAPDH is the enzyme that catalyzes the sixth step of glycolysis, a two-step conversion of glyceraldehyde 3-phosphate into 1,3-bisphosphoglycerate. Galvan-Pena et al. [107] demonstrated that, in LPS-stimulated macrophages, GAPDH undergoes malonylation, which increases its enzymatic activity and enables the production of pro-inflammatory cytokines, such as IL-1β and IL-6. The use of koningic acid [1, 5, 10 μM] reduced the transcription of IL-1β, IL-6, and TNF-α. Also, the protein levels were downregulated, indicating the crucial role of GAPDH in macrophage activation [107]. Similar results, verifying the importance of GAPDH action in macrophages, were presented by Liao et al. [71]. The described phenomenon has also been recently explored by Zhang et al. [108]. The authors used koningic acid (heptelidic acid, HA) to verify whether enhanced glycolysis in macrophages supports HIV-1-induced M1 polarization. The presented data indicated that human HIV-1-infected macrophages release a high amount of pro-inflammatory cytokines, including IL-1β, IL-6, and TNF-α, but the presence of koningic acid [10 μM] significantly downregulated their levels [108].

Due to its potent proapoptotic action, koningic acid has been tested in various cancers [109,110]. Further, it was reported to prevent abnormal Tau protein aggregation in an Alzheimer’s disease model [111]. However, its anti-inflammatory potential in various autoimmunity models is yet to be explored.

Table 1 briefly summarizes the above-described glycolysis inhibitors, their intracellular targets, and experimental models used for the verification of their biological action.

## 10. Conclusions and Perspectives

**Figure 4:**
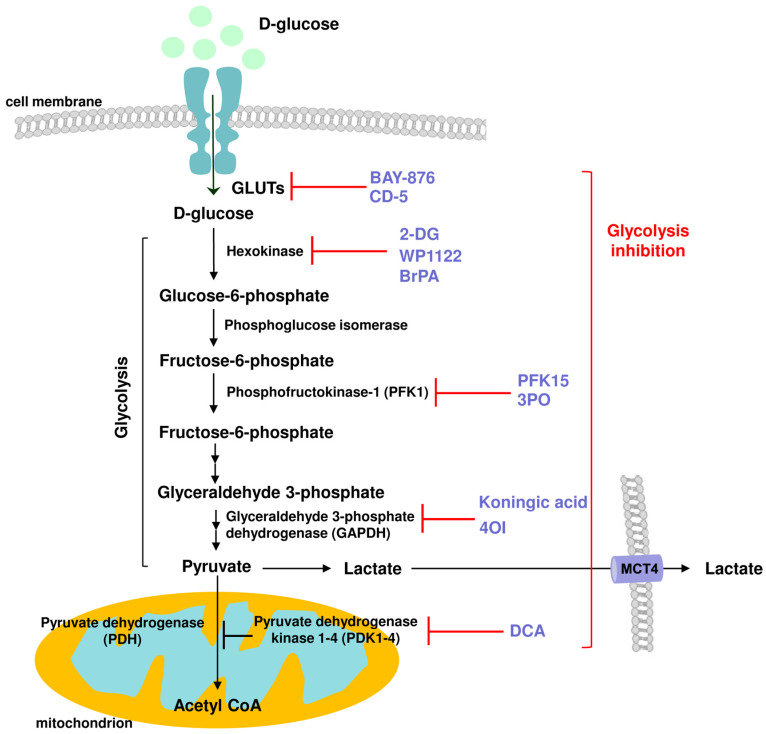
Schematic representation of the action of various glycolysis inhibitors and their cellular targets: GLUTSs: BAY-876, CD-5; Hexokinase: 2-DG, WP1122, BrPA; Phosphofructokinase 1 (PFK1): PFK15, 3PO; Glyceraldehyde 3-phosphate dehydrogenase (GAPDH): koningic acid, 4-OI; Pyruvate dehydrogenase kinase (PDK): DCA, with anti-inflammatory potential.

A growing body of evidence supports the crucial importance of metabolic shift and glycolysis upregulation during inflammatory processes. Aside from immune responses to infection, inflammatory processes are a common denominator for many age-associated diseases, such as cardiovascular and neurodegenerative disorders. Their uncontrolled activation is also responsible for multiple autoimmune diseases. The finding that immuno-metabolism could be a master regulator of inflammation opens new doors for potential therapeutic interventions targeting inflammation-related pathologies through cellular metabolism, especially the inhibition of glycolysis. This strategy has been effective in targeting glycolysis-elevated cancer cells and several glycolysis inhibitors have undergone pre-clinical testing and even been introduced into clinical trials. However, the application of glycolysis inhibitors, with regard to treating hyper-inflammation and autoimmunity, is not fully explored. A summary of the compounds that were described in this review article due to their ability to restrain glycolysis activity and exert biological effects is presented in Figure 4.

We believe that targeting glycolysis could be a potent and universal autoimmune strategy and we are going to further explore the above concept in our future research.

## Figures and Tables

**Table 1:** Glycolysis inhibitors and their anti-inflammatory activity.

Inhibitor	Intracellular Target	Biological Action	Refs.
BAY-876	GLUTs	↓ CD4+ T cells ↓ macrophages	[103]
CD-5	GLUTs	↓ CD4+ T cells ↓ Th1 and TH17 polarization ↑ T_reg_ differentiation	[104,105]
2-DG	Hexokinase	↓ Macrophages polarization (asthma model and chitin-induced inflammation) ↓ Peritoneal macrophages (adjuvant arthritis model) ↓ IL-6 signaling pathway (bowel disease and rheumatoid arthritis models) ↓ LPS-induced pulmonary inflammation	[33,34,35,42]
WP1122	Hexokinase	↓ Glycolysis (cancer models)	[69,70]
BrPA	Hexokinase	↓ Th_17_ cells ↓ DCs activation ↑ T_reg_ differentiation (rheumatoid arthritis model)	[102]
PFK15	PFK1	↓ T cells metabolic reprogramming (type 1 diabetes model) ↓ LPS-induced DCs cells ↑ Immunosuppressive T cells (CIA model) ↓ CD45+ cells, chemokines (IBD model)	[77,78,79]
3PO	PFK1	↓ LPS-stimulated activation of human alveolar epithelial cells ↓ Pro-inflammatory cytokines, ROS (sepsis-induced lung injury model) ↓ TLR2-induced NF-κB, STAT3 activation, pro-inflammatory cytokines (rheumatoid arthritis model) ↓ Atherosclerotic macrophages ↓ Synovia inflammation	[84,85,86,87,88]
Koningic acid	GAPDH	↓ LPS-stimulated macrophages ↓ M1 macrophages polarization ↓ Pro-inflammatory cytokines (HIV-infected macrophages)	[107,108]
4-OI	GAPDH	↓ LPS-stimulated macrophages and IL-1β secretion ↓ CD4+ and CD8+ T cells	[71,72,73]
DCA	PDK	↓ CIA-induced arthritis ↓ Sepsis-induced inflammation ↓ Atherosclerosis ↓ Colitis	[93,94,95,96]

↑—upregulation, ↓—downregulation.

## Data Availability

Not applicable.
